# Nuclear receptor coregulator SNP discovery and impact on breast cancer risk

**DOI:** 10.1186/1471-2407-9-438

**Published:** 2009-12-14

**Authors:** Ryan J Hartmaier, Sandrine Tchatchou, Alexandra S Richter, Jay Wang, Sean E McGuire, Todd C Skaar, Jimmy M Rae, Kari Hemminki, Christian Sutter, Nina Ditsch, Peter Bugert, Bernhard HF Weber, Dieter Niederacher, Norbert Arnold, Raymonda Varon-Mateeva, Barbara Wappenschmidt, Rita K Schmutzler, Alfons Meindl, Claus R Bartram, Barbara Burwinkel, Steffi Oesterreich

**Affiliations:** 1 Program in Translational Biology & Molecular Medicine, Baylor College of Medicine, 1 Baylor Plaza, Houston TX, 77030, USA; 2 Lester & Sue Smith Breast Center, Baylor College of Medicine, 1 Baylor Plaza, Houston TX, 77030, USA; 3 Division Molecular Biology of Breast Cancer, Department of Gynecology and Obstetrics, University of Heidelberg, Voßstrasse9, 69115 Heidelberg, Germany; 4 Helmholtz-University Group Molecular Epidemiology, German Cancer Research Center (DKFZ), Im Neuenheimer Feld 581, 69120 Heidelberg, Germany; 5 Martin-Luther-Universitaet Halle-Wittenberg, Universitätsplatz 10, 06108 Halle, Germany; 6 Division of Clinical Pharmacology, Indiana University School of Medicine, 1120 South Drive, Indianapolis, IN, 46202, USA; 7 University of Michigan, 1150 W Medical Center Drive, Ann Arbor, MI, 48109, USA; 8 Division of Molecular Genetic Epidemiology, German Cancer Research Center (DKFZ), Im Neuenheimer Feld 580, Germany, 69120 Heidelberg, Germany; 9 Department of Biosciences at Novum, Karolinska Institute, 14157 Huddinge, Sweden; 10 Institute of Human Genetics, University of Heidelberg, Im Neuenheimer Feld 366, 69120 Heidelberg, Germany; 11 Department for Obstetrics and Gynaecology, Ludwig Maximilians Universität, Marchioninistr 15, 81377 Munich, Germany; 12 Institute of Transfusion Medicine and Immunology, Red Cross Blood Service of Baden-Württemberg-Hessen, University of Heidelberg, Medical Faculty of Mannheim, Friedrich-Ebert-Str 107, 68167 Mannheim, Germany; 13 Institute of Human Genetics, University of Regensburg, Franz-Josef-Strauss-Allee 11, 93053 Regensburg, Germany; 14 Division of Molecular Genetics, Department of Gynaecology and Obstetrics, Clinical Center University of Düsseldorf, 40225 Düsseldorf, Germany; 15 Division of Oncology, Department of Gynaecology and Obstetrics, University Hospital Schleswig-Holstein, Arnold-Heller-Str 3, 24105 Kiel, Germany; 16 Institut für Humangenetik, Charité - Universitätsmedizin Berlin, Augustenburger Platz 1, 13353 Berlin, Germany; 17 Division of Molecular Gyneco-Oncology, Department of Gynaecology and Obstetrics, Center of Molecular Medicine Cologne (CMMC), University Hospital of Cologne, Kerpener Straße 34, 50931 Cologne, Germany; 18 Division of Gynecology and Obstetrics, Klinikum rechts der Isar at the Technical University Munich, 80336 Munich, Germany; 19 Departments of Medicine, Baylor College of Medicine, 1 Baylor Plaza, Houston TX, 77030, USA; 20 Molecular & Cellular Biology, Baylor College of Medicine, 1 Baylor Plaza, Houston TX, 77030, USA

## Abstract

**Background:**

Coregulator proteins are "master regulators", directing transcriptional and posttranscriptional regulation of many target genes, and are critical in many normal physiological processes, but also in hormone driven diseases, such as breast cancer. Little is known on how genetic changes in these genes impact disease development and progression. Thus, we set out to identify novel single nucleotide polymorphisms (SNPs) within SRC-1 (NCoA1), SRC-3 (NCoA3, AIB1), NCoR (NCoR1), and SMRT (NCoR2), and test the most promising SNPs for associations with breast cancer risk.

**Methods:**

The identification of novel SNPs was accomplished by sequencing the coding regions of these genes in 96 apparently normal individuals (48 Caucasian Americans, 48 African Americans). To assess their association with breast cancer risk, five SNPs were genotyped in 1218 familial BRCA1/2-mutation negative breast cancer cases and 1509 controls (rs1804645, rs6094752, rs2230782, rs2076546, rs2229840).

**Results:**

Through our resequencing effort, we identified 74 novel SNPs (30 in NCoR, 32 in SMRT, 10 in SRC-3, and 2 in SRC-1). Of these, 8 were found with minor allele frequency (MAF) >5% illustrating the large amount of genetic diversity yet to be discovered. The previously shown protective effect of rs2230782 in SRC-3 was strengthened (OR = 0.45 [0.21-0.98], p = 0.04). No significant associations were found with the other SNPs genotyped.

**Conclusions:**

This data illustrates the importance of coregulators, especially SRC-3, in breast cancer development and suggests that more focused studies, including functional analyses, should be conducted.

## Background

Nuclear receptors are critical for proper development and function of many physiological pathways including lipid metabolism, inflammation, and cell growth [1-3]. Over the past 25 years, it has become clear that nuclear receptors are also critical for the onset and progression of many diseases, including cancer. In breast cancer, for example, estrogen receptor-α (ERα) is expressed and drives tumor growth in approximately 2/3 of cases. However, only recently it has been appreciated that proper nuclear receptor function is absolutely dependent on the interaction with coregulator proteins [4]. These proteins couple nuclear receptors with RNA polymerase II and chromatin remodeling machinery to either activate (coactivators) or repress (corepressors) nuclear receptor mediated gene transcription. And because a single or a subset of coregulators can simultaneously regulate multiple cellular processes through multiple nuclear receptors, they have been classified as 'master regulators' [3]. Keeping with this classification, many coregulators have been implicated in numerous human diseases, including breast cancer [5-10].

Family history is one of the strongest risk factors for breast cancer with the risk approximately double in first degree relatives of women with breast cancer compared to the general population [11]. Because of this, many attempts to identify genetic risk factors using multiple approaches have been conducted. However, despite the identification of mutations in the major risk factor genes such as BRCA1, BRCA2, PTEN, CHEK2, and ATM, it is estimated that ~75% of familial breast cancers have yet unidentified risk alleles [12]. ERα is expressed and drives a large fraction of breast cancer cases and is therefore an excellent candidate gene for identifying breast cancer risk factors. Recently, a significant association with familial breast cancer risk has been observed for the C allele of ESR1_rs2747648 in an allele dose-dependent manner. This variant is located in a miRNA-binding site in the 3' untranslated region of ESR1 [13]. However, historically very few associations have been found between SNPs in ERα and breast cancer risk. Further, a recent study conducted a comprehensive search of all SNPs in ERα that revealed no major risk associations (n>55,000 breast cancer cases and controls) [14]. This suggests that other players in the ER signaling pathway may be important for breast cancer risk. Because of the critical importance of coregulators for ERα function, we hypothesized that breast cancer risk is influenced by SNPs within the coactivators SRC-1/NCoA1 and SRC-3/NCoA3/AIB1 and the corepressors NCoR and SMRT/NCoR2.

We previously reported two SNPs in SRC-3 (rs2230782 and rs2076546) associated with reduced breast cancer risk in a case-control study of German and Polish high-risk, BRCA1/2 mutation-negative women (cases: 775, controls: 1628) [15]. In a recent study by Haiman et al [16], coregulator sequencing was conducted in 95 women with advanced breast cancer from the Multiethnic Cohort (African Americans, Latinos, Japanese, Native Hawaiians, and European Americans) to identify novel SNPs and determine their contribution to breast cancer risk in the Multiethnic Cohort (cases: 1612, controls: 1961). Two SNPs were significantly associated with breast cancer risk in this study (one in each of SMRT and CALCOCO1). These SNPs, however, are found exclusively or nearly exclusively in African Americans and therefore cannot be feasibly tested in DNA banks derived from European individuals. One SRC-3 SNP previously identified to be protective in our study [15] was genotyped (rs2230782) and found not to be associated with altered breast cancer risk in the Haiman study [16]. The other SNP we reported to be protective (rs2076546) was not genotyped in this study since it focused on non-synonymous SNPs.

Here we report an extension of our previous study that identified two SNPs within SRC-3 associated with reduced breast cancer risk [15]. We followed a similar approach by genotyping candidate SNPs for associations with breast cancer risk in a high-risk, BRCA1/2 mutation-negative case-control study; however, the original study was extended in three ways. First, three additional coregulators were examined. Second, we sequenced 96 apparently normal individuals from two populations (48 Caucasian Americans and 48 African Americans) to discover novel SNPs and to confirm or reveal SNP frequency information in different populations. Third, a larger population was examined, almost doubling the number of cases and significantly improving our statistical power. The association studies allowed us to strengthen the significance of the protective effect previously reported for a SNP in SRC-3 while extending it to a rare two-SNP haplotype that is highly protective for breast cancer risk.

## Methods

### SNP Discovery

Target sequence obtained from NCBI consisting of all exons, 500 bp of proximal promoter, and 25 bp of flanking introns from SRC-1, SRC-3, NCoR, and SMRT was submitted for primer design and Sanger sequencing to Polymorphic DNA Technologies Inc. (Alameda CA). DNA from 96 samples (48 Caucasian American, 48 African American) obtained from the Coriell Institute (Camden, NJ, USA) (sample sets: HD100CAU and HD100AA) was sequenced in both directions and aligned to NCBI reference sequence and previously reported SNPs in dbSNP. These samples had been collected and anonymized by the National Institute of General Medical Sciences. Visual inspection of chromatograms was conducted for heterozygous calls.

### Genotyping Cohort

A case-control study was conducted investigating a German familial breast cancer study cohort. Unrelated, German, female BRCA1/2 mutation negative index cases from breast cancer families were used in this study. The samples, all of Caucasian origin, were collected during the years 1997-2005 by six centers of the German Consortium for Hereditary Breast and Ovarian Cancer (GC-HBOC: centers of Heidelberg, Würzburg, Cologne, Kiel, Düsseldorf and Munich, see authors affiliations). Familial cases were identified based on (A1) families with two or more breast cancer cases including at least two cases with onset below the age of 50 years; (A2) families with at least one male breast cancer case; (B) families with at least one breast cancer and one ovarian cancer case; (C) families with at least two breast cancer cases including one case diagnosed before the age of 50 years; (D) families with at least two breast cancer cases diagnosed after the age of 50 years; (E) single cases of breast cancer with age of diagnosis before 35 years. These selection criteria which have previously been reported [17] enrich for cases caused by genetic factor(s). The control population included healthy and unrelated female blood donors collected by the Institute of Transfusion Medicine and Immunology (Mannheim), sharing the ethnic background and sex with the breast cancer patients. The age distribution in the controls and cases was similar (controls: mean age 45.6 years, median age 46 years, age range from 18 to 68 years old; cases: mean age 45.1 years, median age 45 years, age range from 19 to 87 years old). According to the German guidelines for blood donation, all blood donors were examined by a standard questionnaire and gave their informed consent. They were randomly selected during the years 2004-2007 for this study and no further inclusion criteria were applied during recruitment. The study was approved by the Ethics Committee of the University of Heidelberg (Heidelberg, Germany).

### Genotyping

Genotyping was conducted using TaqMan allelic discrimination assays. Primers and TaqMan MGB probes were purchased from Applied Biosystems (Foster City, CA).

SRC-3 Q586H: 5'-CTGGGCTTTTATTGCGACCAAA-3V, reverse 5VGCTCTCCTTACTTTCTTTGTCACTGA-3'; TaqMan probes: forward 5'-TTCAATGTGTCACTCAAAT-3'-VIC, reverse 5'-CAATGTGTCAGTCAAAT-3'-FAM.

SRC-3 T960T: forward 5'-CCTGCACTGGGTGGCT-3', reverse 5'-CTCGCACCTGGTATGCTATTAGAC-3'; TaqMan probes: forward 5'-CTATTCCCACATTGCCTC-3'-VIC, reverse 5'-TTCCCACGTTGCCTC-3'-FAM.

SRC-3 C218R: forward 5'-AGACATAAACGCCAGTCCTGAAATG-3', reverse 5'-GCCAGAGATATGAAACAATGCAGTG-3'; TaqMan probes: forward 5'-TGAAATGCGCCAGAG-3'-VIC, reverse 5'-TGAAATGTGCCAGAG-3'-FAM.

SRC-1 P1272S: forward 5'-CCCTCCTCCTCAGAGTTCTCT-3', reverse 5'-CCTTCATGTCTGGTGACTGATACC-3'; TaqMan probes: forward 5'-CAGGTGGAGTTTGC-3'-VIC, reverse 5'-CAGGTGAAGTTTGC-3'-FAM.

SMRT A1706T: forward 5'-ACCTCGCAGCAGATGCA-3', reverse 5'-GAGGCCCCTCAGCATATCAG-3'; TaqMan probes: forward 5'-CCACAACACGGCCAC-3'-VIC, reverse 5'-CACAACGCGGCCAC-3'-FAM.

Genotyping call rates for all studies were >97%. The SNP assays were validated by re-genotyping 5% of all samples. The concordance rate for all SNPs varied from 99 to 100%.

### Statistical Analysis

Hardy-Weinberg equilibrium test was undertaken using the chi-square "goodness-of-fit" test. Crude odds ratios (ORs), 95% confidence intervals (95% CIs) and *P *values were computed by unconditional logistic regression using a tool offered by the Institute of Human Genetics, Technical University Munich, Germany http://ihg.gsf.de/cgi-bin/hw/hwa1.pl. Power calculations were determined using power and sample size calculator software PS version 2.1.31 http://www.mc.vanderbilt.edu/prevmed/ps/. With the total sample size, we had 80% power to detect OR of 0.79/1.26 and 0.57/1.56 for carrier frequencies of 30% and 5%, respectively.

### Haplotype Analysis

Haplotypes of variants located in the same gene were determined using the PHASE 2 software created by Stephens *et al*. [18], or SNPHAP 1.3 software created by David Clayton http://www-gene.cimr.cam.ac.uk/clayton/software/snphap.txt. Each individual was assumed to carry the most likely pair of haplotypes and the haplotype distributions were estimated based on the controls.

## Results/Discussion

### SNP Discovery

**Figure 1:**
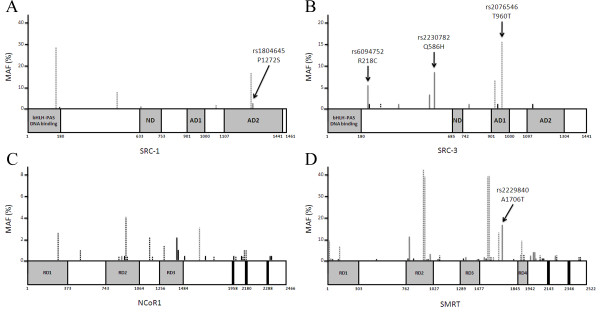
**SNP discovery in (A) SRC-1, (B) SRC-3, (C) NCoR, and (D) SMRT**. Vertical lines delineate the position of SNPs identified by our resequencing effort. The height of the vertical lines represents the frequency at which the SNP was found. Black lines represent novel SNPs, grey lines represent SNPs found in dbSNP. Solid lines represent nonsynonymous SNPs, dashed lines represent synonymous SNPs. Positions of SNPs genotyped for risk associations are pointed out by arrows.

Complete coding regions and 25 bp of the flanking intronic regions of SRC-1, SRC-3, NCoR, and SMRT were fully sequenced in both directions using Sanger sequencing in 96 apparently normal individuals (48 Caucasian American, 48 African American) generating a total of ~5.8 MB of sequence. From this effort we identified 120 SNPs (61 in SMRT, 33 in NCoR, 18 in SRC-3, and 8 in SRC-1). A summary of the results is shown in Table 1 and details are provided in Additional File 1. Of these, 86 coding SNPs were identified resulting in 36 nonsynonymous SNPs (nsSNPs). SMRT contained the largest number of SNPs (61 total, 43 coding, and 17 nsSNPs). Despite its close relationship with SMRT, NCoR contains far fewer SNPs (33 total, 25 coding, and 10 nsSNPs). This is especially evident when only common SNPs are considered (minor allele frequency [MAF]>5%; 16 in SMRT, 1 in NCoR). The position of the coding SNPs and the MAF is schematically presented in Figure 1.

**Table 1:** SNP Discovery Summary.

Gene	Total SNPs	Coding SNPs	nsSNPs	Total SNPs MAF>5%	Novel SNPs	Novel nsSNPs	Novel SNPs MAF>5%
SMRT	61	43	17	16	32	6	7
NCoR	33	25	10	1	30	9	0
SRC-3	18	11	8	5	10	3	1
SRC-1	8	7	1	3	2	0	0

Total	120	86	36	25	74	18	8

By conducting the sequencing in two populations, we were able to distinguish SNPs unique to a particular population. We identified 66 SNPs unique to African Americans and 23 SNPs unique to Caucasian Americans (see Additional File 1). This distribution is similar to that reported previously in the SNP@Ethnos database for Yoruban and European populations and is hypothesized to arise from bottlenecks in non-African population history[19] However, most of the unique SNPs found in Caucasians were rare, possibly suggesting that these are recent alterations since only 4 out of the 23 unique SNPs (17%) were found in more than a single individual. On the other hand, 31 out of the 66 unique SNPs (47%) in African Americans were found in more than a single individual. It is important to note that some of the population unique SNPs are rare and since only 48 individuals were sequenced for each population, they could appear as unique SNPs purely by chance.

From our sequencing effort we identified 74 SNPs in these four coregulators not previously represented in dbSNP or reported in the recent study by Haiman et al [16] (Table 1, columns on the right). We will refer to these SNPs as novel SNPs. Surprisingly, 8 of these novel SNPs were found at MAF>5% (7 within SMRT and 1 within SRC-3). Of the 74 novel SNPs, 18 were nonsynonymous, again with SMRT harboring many of the alterations. This illustrates that SMRT is by far the most polymorphic of the 4 coregulators. A recent study suggests that mutation rate, compared to selection pressure, has a larger impact on polymorphism frequency in a region [20]. Further, areas of condensed chromatin have been suggested to have the highest level of background mutation [21]. Together this suggests that SMRT is under less selective pressure than NCoR and/or is in a region of the genome with a higher mutation rate (possibly in an area of condensed chromatin).

### Genotyping for Association with Breast Cancer Risk

We genotyped a case-control study of female index patients of BRCA1/BRCA2 mutation negative breast cancer families for two SNPs in SRC-3 which were previously shown to have a protective effect for breast cancer [15] (rs2230782 and rs2076546). Additionally, we genotyped other coregulator SNPs we rationalized may have functional consequences based on the severity of the amino acid change and proximity to functional domains [rs1804645 (SRC-1), rs6094752 (SRC-3), and rs2229840 & rs7978237 (SMRT)] (positions are highlighted in Figure 1). For example, rs1804645 (SRC-1 P1272S) was chosen since it is the only non-synonymous SNP in SRC-1, is located in the second activation domain, and is predicted to be 'probably damaging' by a polymorphism phenotype prediction tool (PolyPhen, http://genetics.bwh.harvard.edu/pph/). Rs6094752 (SRC-3 R218C) was chosen because of the loss of charge and size as a result of the amino acid substitution, and is one of the most common non-synonymous SNPs in SRC-3. The SNPs in SMRT, rs2229840 (A1706T) and rs7978237 (G781E) were chosen for genotyping due to high frequency, severity of amino acid change, and location in a functional domain. Several approaches to design TaqMan assays for rs7978237 failed. We were therefore unable to obtain genotyping information for this SNP.

The genotyping results were in Hardy-Weinberg equilibrium in controls for all SNPs investigated (p = 0.309 for rs1804645; p = 0.112 for rs6094752; p = 0.058 for rs2230782; p = 0.067 for rs2076546; p = 0.140 for rs2229840). The three SNPs that we rationalized may have functional consequences that we were able to genotype, namely SRC-1 P1272S (rs1804645), SRC-3 R218C (rs6094752), and SMRT A1706T (rs2229840), did not significantly associate with breast cancer risk (Table 2). Also, stratification for age (> = 50 year and <50 years of age) in order to investigate a possible risk influence in pre- or postmenopausal women revealed no significant associations except for rs6094752 where a significant effect could be detected for heterozygous carriers only (Table 3). However, this is most likely a chance effect due to multiple testing. Stratification by bilateral cases revealed no significant associations (Table 4). We observed a protective effect of the homozygous c-allele carrier of SRC-3 Q586H rs2230782 (GG+GC versus CC: OR = 0.45, 95%CI = 0.041, Table 2), similar to the findings that have been reported before (GG+GC versus CC: OR = 0.39, 95%CI = 0.14-1.05 p = 0.061) [15]. As our study included a portion of the samples of the previous reported study it is noteworthy to mention that the results of the current study excluding the previously analyzed samples show the same protective effect and borderline significance (GG+GC versus CC: OR = 0.37, 95%CI = 0.13-1.08, p = 0.059). However, we failed to replicate previous associations between SRC-3 rs2076546 (T960T) SNP and breast cancer risk. The haplotype analysis of the variants analysed in SRC-3 revealed a protective haplotype including the C-C-G-alleles of R218C, Q586H and T960T, respectively (Table 5). As the haplotype is very rare occurring with a frequency of 0.03 in controls this result has to be verified in further multi-center collaboration studies.

**Table 2:** Summary of associations in entire population

SNP	Genotypes	Cases	Controls	OR	95% CI	*P*
**SRC-1**	CC (%)	1147 (94.2)	1432 (94.9)	1		
**rs1804645**	CT (%)	69 (5.6)	77 (5.1)	1.11	0.80-1.56	0.510
***P1272S***	TT (%)	2 (0.2)	0 (0.0)	-	-	-
		[CT + TT]<-> [CC]		1.15	0.82-1.60	0.405
**SRC-3**	CC (%)	1089 (89.9)	1330 (89.3)	1		
**rs6094752**	CT (%)	116 (9.6)	152 (10.2)	0.93	0.72-1.20	0.587
***C218R***	TT (%)	6 (0.5)	8 (0.5)	0.92	0.32-2.65	0.871
		[CT + TT]<-> [CC]		0.93	0.73-1.19	0.575
**SRC-3**	GG (%)	988 (80.8)	1207 (80.3)	1		
**rs2230782**	CG (%)	226 (18.5)	272 (18.1)	1.11	0.83-1.23	0.881
***Q586H***	CC (%)	9 (0.7)	24 (1.6)	**0.46**	**0.21-0.99**	**0.042**
		[CC]<-> [GG + GC]		0.45*	0.21-0.98*	0.041*
**SRC-3**	AA (%)	1011 (82.7)	1240 (82.2)	1		
**rs2076546**	AG (%)	202 (16.5)	249 (16.5)	0.99	0.25-1.22	0.961
***T960T***	GG (%)	9 (0.8)	20 (1.3)	0.55	0.25-1.22	0.135
		[AG + GG]<-> [AA]		0.96°	0.79-1.17°	0.702°
**SMRT**	GG (%)	789 (66.2)	1004 (67.6)	1		
**rs2229840**	AG (%)	357 (30.0)	423 (28.5)	1.07	0.91-1.27	0.407
***A1706T***	AA (%)	45 (3.8)	57 (3.9)	1.01	0.67-1.50	0.982
		[AG + GG]<-> [AA]		1.06	0.91-1.25	0.441

Odds ratios (OR) with 95% confidence intervals (95% CI) and *P*-values. Please note that the results excluding samples (345 cases/1190 controls) that have been analysed in a previous study (14) are *OR = 0.37, 95%CI = 0.13-1.08, p = 0.059 and °OR = 1.13, 95%CI = 0.80-1.59, p = 0.491.

**Table 3:** Associations according to age stratification

SNP	Genotypes	Cases	Controls	OR	95% CI	P
**Cases and Controls ≥50**						

**SRC-1**	CC (%)	306 (93.9)	747 (94.9)	1		
**rs1804645**	CT (%)	20 (6.1)	40 (5.1)	1.22	0.70-2.12	0.479
***P1272S***	TT (%)	0 (0.0)	0 (0.0)	-	-	-
		[CT + TT]<-> [CC]		1.22	0.70-2.12	0.479
**SRC-3**	CC (%)	299 (92.8)	691 (89.0)	1		
**rs6094752**	CT (%)	20 (6.2)	80 (10.4)	**0.58**	**0.35-0.96**	**0.033**
***C218R***	TT (%)	3 (1.0)	5 (0.6)	1.39	0.33-5.84	0.654
		[CT + TT]<-> [CC]		0.62	0.39-1.01	0.053
**SRC-3**	GG (%)	253 (78.1)	623 (79.4)	1		
**rs2230782**	GC (%)	68 (21.0)	150 (19.1)	1.12	0.81-1.54	0.502
***Q586H***	CC (%)	3 (0.9)	12 (1.5)	0.62	0.17-2.20	0.451
		[CC]<-> [GG + GC]		0.60	0.16-2.14	0.429
**SRC-3**	AA (%)	270 (83.6)	653 (82.5)	1		
**rs2076546**	AG (%)	52 (16.1)	126 (15.9)	0.99	0.70-1.42	0.991
***T960T***	GG (%)	1 (0.3)	12 (1.6)	0.20	0.02-1.56	0.088
		[AG + GG]<-> [AA]		0.93	0.66-1.31	0.676
**SMRT**	GG (%)	222 (69.4)	517 (67.0)	1		
**rs2229840**	AG (%)	89 (27.8)	220 (28.5)	0.94	0.70-1.26	0.689
***A1706T***	AA (%)	9 (2.8)	35 (4.5)	0.59	0.28-1.27	0.175
		[AG + GG]<-> [AA]		0.89	0.68-1.18	0.439

**Cases and Controls < 50**						

**SRC-1**	CC (%)	682 (94.4)	685 (94.9)	1		
**rs1804645**	CT (%)	38 (5.3)	37 (5.1)	1.03	0.65-1.64	0.896
***P1272S***	TT (%)	2 (0.3)	0 (0.0)	-	-	-
		[CT + TT]<-> [CC]		1.08	0.69-1.72	0.725
**SRC-3**	CC (%)	633 (88.6)	639 (89.4)	1		
**rs6094752**	CT (%)	77 (10.8)	72 (10.1)	1.08	0.77-1.52	0.658
***C218R***	TT (%)	4 (0.6)	4 (0.6)	1.01	0.25-4.05	0.989
		[CT + TT]<-> [CC]		1.08	0.77-1.50	0.665
**SRC-3**	GG (%)	592 (82.2)	584 (81.3)	1		
**rs2230782**	GC (%)	122 (16.9)	122 (17.0)	0.98	0.75-1.30	0.923
***Q586H***	CC (%)	6 (0.9)	12 (1.7)	0.49	0.18-1.32	0.152
		[CC]<-> [GG + GC]		0.49	0.18-1.32	0.153
**SRC-3**	AA (%)	592 (82.1)	587 (81.7)	1		
**rs2076546**	AG (%)	123 (17.1)	123 (17.1)	0.99	0.75-1.30	0.952
***T960T***	GG (%)	6 (0.8)	8 (1.2)	0.74	0.26-2.16	0.584
		[AG + GG]<-> [AA]		0.98	0.75-1.28	0.862
**SMRT**	GG (%)	470 (66.9)	487 (68.4)	1		
**rs2229840**	AG (%)	202 (28.8)	203 (28.5)	1.03	0.82-1.30	0.796
***A1706T***	AA (%)	30 (4.3)	22 (3.1)	1.41	0.80-2.48	0.228
		[AG + GG]<-> [AA]		1.07	0.85-1.33	0.561

Odds ratios (OR) with 95% confidence intervals (95% CI) and *P*-values.

**Table 4:** Associations with stratification by bilateral cases

SNP	Genotypes	Cases	Controls	OR	95% CI	P
**Cases bilateral**						

**SRC-1**	CC (%)	106 (93.8)	1432 (94.9)	1		
**Rs1804645**	CT (%)	6 (5.3)	77 (5.1)	1.05	0.44-2.47	0.906
***P1272S***	TT (%)	1 (0.9)	0 (0.0)	-	-	-
		[CT + TT]<-> [CC]		1.22	0.55-2.73	0.613
**SRC-3**	CC (%)	95 (86.4)	1330 (89.3)	1		
**rs6094752**	CT (%)	14 (12.7)	152 (10.2)	1.29	0.72-2.32	0.393
***C218R***	TT (%)	1 (0.9)	8 (0.5)	1.75	0.22-14.14	0.595
		[CT + TT]<-> [CC]		1.31	0.74-2.32	0.347
**SRC-3**	GG (%)	88 (80.0)	1207 (80.3)	1		
**Rs2230782**	GC (%)	22 (20.0)	272 (18.1)	1.11	0.68-1.80	0.675
***Q586H***	CC (%)	0 (0.7)	24 (1.6)	0.28	0.017-4.62	0.186
		[CC]<-> [GG + CT]		1.02	0.63-1.65	0.938
**SRC-3**	AA (%)	89 (80.2)	1240 (82.2)	1		
**rs2076546**	AG (%)	21 (18.9)	249 (16.5)	1.17	0.72-1.93	0.522
***T960T***	GG (%)	1 (0.9)	20 (1.3)	0.69	0.09-5.25	0.724
		[AG + GG]<-> [AA]		1.14	0.70-1.85	0.597
**SMRT**	GG (%)	79 (72.5)	1004 (67.6)	1		
**rs2229840**	AG (%)	27 (24.8)	423 (28.5)	0.81	0.52-1.27	0.363
***A1706T***	AA (%)	3 (2.7)	57 (3.9)	0.67	0.20-2.18	0.502
		[AG + GG]<-> [AA]		0.79	0.51-1.23	0.298

Odds ratios (OR) with 95% confidence intervals (95% CI) and *P*-values.

**Table 5:** Associations of SRC-3 haplotypes

Haplotypes^a^	Cases (%)	Controls (%)	OR	95% CI	*P*
**CGA**	1847 (77.0)	2199 (75.0)	1		
CCA	227 (9.5)	298 (10.1)	0.91	0.75-1.09	0.2963
CGG	205 (8.5)	267 (9.1)	0.91	0.75-1.11	0.3597
TGA	119 (4.9)	162 (5.5)	0.87	0.68-1.12	0.2824
CCG	0 (0.0)	8 (0.3)	**0.07**	**0.004-1.21**	**0.0096**
TCG	2 (0.1)	1 (0.0)	2.38	0.22-26.28	0.4651
TGG	0 (0.0)	1 (0.0)	0.39	0.02-9.74	0.3659

^a ^haplotypes representing the alleles of C218R, Q586H and *T960T *compared with wildtype. Haplotype frequencies of SRC-3 polymorphisms C218R, Q586H and T960T in the German study population compared with the, odds ratios (OR) with 95% confidence intervals (95% CI) and *P*-values.

The discordant findings between our studies and the Haiman study [16] with respect to SRC-3 Q586H may be due to the inherent differences in the populations examined. For example, our studies exclusively examined Europeans while the study by Haiman et al. examined a range of ethnic backgrounds. A number of recent studies suggest that a SNP association could be specific to the genetic background of a certain ethnic group [22,23]. It is possible that the Q586H effect is only seen in European populations, and/or that the lower number of unselected European cases within the Haiman study had insufficient power to detect this effect. The selection of high risk BRCA1/BRCA2 mutation negative cases in our study is expected to act as a multiplier to further increase our power to detect associations. Lastly, since only nonsynonymous SNPs were genotyped in the Haiman study, the stronger effect seen in the two-SNP SRC-3 haplotype could not be observed. We did not genotype the two SNPs (SMRT H52R and CALCOCO1 R12H) identified in the Haiman study to be associated with breast cancer risk since they were found either exclusively or predominantly in African Americans (European population MAF: SMRT H52R = 0%, CALCOCO1 R12H = 0.6%). Since our study exclusively contains Europeans, it was unlikely that we would obtain sufficient power to detect an association.

## Conclusions

In summary, these results illustrate the dramatic differences in polymorphism frequency that can be seen amongst closely related genes. Further, the fact that so many novel SNPs were identified through our sequencing effort, even common SNPs with MAF>5%, illustrates the huge amount of genetic diversity that has yet to be discovered. Finally, the strengthening of the association between the SRC-3 Q586H SNP and decreased breast cancer risk, and the identification of a rare haplotype within SRC-3 associated with decreased risk, suggest that this information could be used to help identify a subgroup of high-risk women at a more modest risk. However, this remains to be verified prospectively.

## Competing interests

The authors declare that they have no competing interests.

## Authors' contributions

RJH helped to draft the manuscript, analyzed the re-sequencing data, and participated in the study design and coordination. ST performed genotyping assays and was involved in the statistical analysis and manuscript revisions. ASR, JW, SEM, TCS, JMR, SO were involved in the acquisition of the Coriell samples and their sequencing. KH, CS, ND, PB, BHFW, DN, NA, RVM, BW, RKS, AM, CRB, BB were involved in the generation and analysis of the breast cancer case-control study. BB and SO contributed equally to the study by conceiving it, participating in its design, and helping to draft the manuscript. All authors read and approved the manuscript.

## Pre-publication history

The pre-publication history for this paper can be accessed here:

http://www.biomedcentral.com/1471-2407/9/438/prepub

## Supplementary Material

Additional file 1 **SNP Discovery Table**. All variants in SMRT, NCoR, SRC-1, and SRC-3 identified from the sequencing effort are presented in this additional excel file. Variant information includes: genomic position, coding domain position, rs# (if applicable), nucleotide exchange, amino acid position and exchange (if applicable), and frequency in each of the populations. Click here for file

## References

[B1] Lonard DM O'Malley BW The expanding cosmos of nuclear receptor coactivators Cell 2006 125 3 411 414 10.1016/j.cell.2006.04.021 16678083

[B2] O'Malley BW Coregulators: from Whence Came These 'Master Genes' Mol Endocrinol 2007 21 5 1009 13 10.1210/me.2007-0012 17284664

[B3] Lonard DM O'Malley BW Nuclear receptor coregulators: judges, juries, and executioners of cellular regulation Mol Cell 2007 27 5 691 700 10.1016/j.molcel.2007.08.012 17803935

[B4] Lonard DM Lanz RB O'Malley BW Nuclear receptor coregulators and human disease Endocr Rev 2007 28 5 575 587 10.1210/er.2007-0012 17609497

[B5] Agoulnik IU Vaid A Bingman WE Erdeme H Frolov A Smith CL Ayala G Ittmann MM Weigel NL Role of SRC-1 in the promotion of prostate cancer cell growth and tumor progression Cancer Res 2005 65 17 7959 7967 16140968 10.1158/0008-5472.CAN-04-3541

[B6] Chopra AR Louet JF Saha P An J Demayo F Xu J York B Karpen S Finegold M Moore D Absence of the SRC-2 coactivator results in a glycogenopathy resembling Von Gierke's disease Science 2008 322 5906 1395 1399 10.1126/science.1164847 19039140 PMC2668604

[B7] Myers E Fleming FJ Crotty TB Kelly G McDermott EW O'Higgins NJ Hill AD Young LS Inverse relationship between ER-beta and SRC-1 predicts outcome in endocrine-resistant breast cancer British journal of cancer 2004 91 9 1687 1693 15477868 10.1038/sj.bjc.6602156 PMC2409954

[B8] Redmond AM Bane FT Stafford AT McIlroy M Dillon MF Crotty TB Hill AD Young LS Coassociation of estrogen receptor and p160 proteins predicts resistance to endocrine treatment; SRC-1 is an independent predictor of breast cancer recurrence Clin Cancer Res 2009 15 6 2098 2106 10.1158/1078-0432.CCR-08-1649 19276281

[B9] Sakaguchi H Fujimoto J Sun WS Tamaya T Clinical implications of steroid receptor coactivator (SRC)-3 in uterine endometrial cancers The Journal of steroid biochemistry and molecular biology 2007 104 3-5 237 240 10.1016/j.jsbmb.2007.03.007 17532621

[B10] Wang S Yuan Y Liao L Kuang SQ Tien JC O'Malley BW Xu J Disruption of the SRC-1 gene in mice suppresses breast cancer metastasis without affecting primary tumor formation Proc Natl Acad Sci USA 2009 106 1 151 156 10.1073/pnas.0808703105 19109434 PMC2629242

[B11] Breast Cancer Facts & Figures 2007-2008

[B12] Houlston RS Peto J The search for low-penetrance cancer susceptibility alleles Oncogene 2004 23 38 6471 6476 10.1038/sj.onc.1207951 15322517

[B13] Tchatchou S Jung A Hemminki K Sutter C Wappenschmidt B Bugert P Weber BH Niederacher D Arnold N Varon-Mateeva R A variant affecting a putative miRNA target site in estrogen receptor (ESR) 1 is associated with breast cancer risk in premenopausal women Carcinogenesis 2009 30 1 59 64 10.1093/carcin/bgn253 19028706

[B14] Dunning AM Healey CS Baynes C Maia AT Scollen S Vega A Rodriguez R Barbosa-Morais NL Ponder BA Low YL Association of ESR1 gene tagging SNPs with breast cancer risk Human molecular genetics 2009 18 6 1131 1139 10.1093/hmg/ddn429 19126777 PMC2722230

[B15] Burwinkel B Wirtenberger M Klaes R Schmutzler RK Grzybowska E Forsti A Frank B Bermejo JL Bugert P Wappenschmidt B Association of NCOA3 polymorphisms with breast cancer risk Clin Cancer Res 2005 11 6 2169 2174 10.1158/1078-0432.CCR-04-1621 15788663

[B16] Haiman CA Garcia RR Hsu C Xia L Ha H Sheng X Le Marchand L Kolonel LN Henderson BE Stallcup MR Screening and association testing of common coding variation in steroid hormone receptor co-activator and co-repressor genes in relation to breast cancer risk: the Multiethnic Cohort BMC cancer 2009 9 43 10.1186/1471-2407-9-43 19183483 PMC2637888

[B17] Yang R Frank B Hemminki K Bartram CR Wappenschmidt B Sutter C Kiechle M Bugert P Schmutzler RK Arnold N SNPs in ultraconserved elements and familial breast cancer risk Carcinogenesis 2008 29 2 351 355 10.1093/carcin/bgm290 18174240

[B18] Stephens M Smith NJ Donnelly P A new statistical method for haplotype reconstruction from population data Am J Hum Genet 2001 68 4 978 989 10.1086/319501 11254454 PMC1275651

[B19] Park J Hwang S Lee YS Kim SC Lee D SNP@Ethnos: a database of ethnically variant single-nucleotide polymorphisms Nucleic acids research 2007 35 Database D711 715 10.1093/nar/gkl962 17135185 PMC1747186

[B20] Gorlov IP Gorlova OY Amos CI Relative effects of mutability and selection on single nucleotide polymorphisms in transcribed regions of the human genome BMC genomics 2008 9 292 10.1186/1471-2164-9-292 18559102 PMC2442617

[B21] Prendergast JG Campbell H Gilbert N Dunlop MG Bickmore WA Semple CA Chromatin structure and evolution in the human genome BMC evolutionary biology 2007 7 72 10.1186/1471-2148-7-72 17490477 PMC1876461

[B22] Meulenbelt I Chapman K Dieguez-Gonzalez R Shi D Tsezou A Dai J Malizos KN Kloppenburg M Carr A Nakajima M Large Replication Study and Meta-Analyses of Dvwa as an Osteoarthritis Susceptibility Locus in European and Asian Populations Human molecular genetics 2009 19181678 10.1093/hmg/ddp053

[B23] Zhang G Goldblatt J LeSouef PN Does the relationship between IgE and the CD14 gene depend on ethnicity? Allergy 2008 63 11 1411 1417 10.1111/j.1398-9995.2008.01804.x 18925877

